# LncRNA KCNQ1OT1 promotes osteogenic differentiation to relieve osteolysis via Wnt/β-catenin activation

**DOI:** 10.1186/s13578-018-0216-4

**Published:** 2018-03-07

**Authors:** Xuren Gao, Jian Ge, Weiyi Li, Wangchen Zhou, Lei Xu

**Affiliations:** Department of Orthopedics, The Affiliated Hospital of Xuzhou Medical University, 99 Huaihai West Rd., Xuzhou, 221002 Jiangsu People’s Republic of China

**Keywords:** Osteoblast differentiation, Resveratrol, Wnt/β-catenin signaling, lncRNA KCNQ1OT1, mMSCs

## Abstract

**Background:**

Resveratrol (RSV) has been reported to stimulate osteoblast differentiation in which Wnt/β-catenin signaling pathway played a crucial role. However, whether and how RSV activated Wnt/β-catenin pathway in osteogenic differentiation still remained elusive.

**Methods:**

In vivo polymethylmethacrylate (PMMA) particle-induced osteolysis (PIO) mouse model and in vitro PMMA particle-stimulated mouse mesenchymal stem cells (mMSCs) experiments were established. Relative expression levels of lncRNA KCNQ1OT1, β-catenin, Runx2, Osterix and osteocalcin were determined using quantitative Real-Time PCR. Western blotting was used to measure β-catenin protein expression. In addition, the alkaline phosphatase activity and mineral deposition level using alizarin red S staining were performed to examine osteogenic differentiation status. The interaction between KCNQ1OT1 and β-catenin was confirmed by RNA pull down assay.

**Results:**

RSV significantly attenuated PIO in vivo and PMMA-particle inhibition of osteogenic differentiation of mMSCs. Moreover, KCNQ1OT1 exerted the similar function in mMSCs by regulating β-catenin. Further study demonstrated that RSV exerted its effect on osteoblastic differentiation by regulating KCNQ1OT1. Consequently, RSV alleviated PMMA-particle inhibition of osteoblastic differentiation via Wnt/β-catenin pathway activation in vivo and in vitro.

**Conclusion:**

RSV accelerated osteoblast differentiation by regulating lncRNA KCNQ1OT1 via Wnt/β-catenin pathway activation, indicating the functional role of RSV in modulating osteogenesis.

## Background

It has been well documented that bone remodeling was supported by dynamic equilibrium between bone resorption and bone formation which were regulated and maintained by osteoblasts throughout lifelong [1]. Imbalance especially induced by inhibition of osteogenic differentiation and osteolysis aggravation would result in pathological bone defects including aseptic loosening of the implant during postoperation of total joint arthroplasty (TJA) [2], osteoporosis [3] as well as rheumatoid arthritis [4] and other bone diseases. Recently, a great deal of studies provided evidence that particulate wear particles were the leading causes of periprosthetic osteolysis, which mainly consisted of PMMA [5], ultra-high molecular weight polyethylene (UHMWPE) [6] and titanium [7]. These particles interfered osteoblast homeostasis, induced inflammatory responses and decreased osteoclast differentiation by stimulating MSCs that were the origin of osteoblasts [8]. Thus, it was extremely urgent to investigate possible mechanisms involving in osteoblastic differentiation and to seek an effective agent for treatment of particles-irritated osteolytic diseases.

Multiple endogenous cytokines and growth factors have been identified to play crucial roles in regulating osteoblast differentiation. For instance, bone morphogenetic protein 2 (BMP2) potentiated osteoblastic differentiation of human bone marrow-derived mesenchymal stem cells (hBM-MSCs) via BMP-2/Smad/Runx2 signaling pathway activation [9]. In addition, insulin-like growth factor 1 (IGF-1) could also promote osteogenesis through ERK and JNK MAPK pathway [10], while transforming growth factor β (TGF-β) inhibited osteoblastic differentiation of mesenchymal pluripotent cells as well as preosteoblasts by mediating MAPK-ERK pathway [11]. Meanwhile, a few signaling pathways participating in osteogenesis have been identified, such as PI3-kinase/Akt signaling [12] and Wnt/β-catenin pathway [13]. Thereinto, Wnt/β-catenin pathway, which functioned as a pivotal regulator of bone homeostasis, was one of the most common targets for interventional therapy of patients with bone fracture [14]. Additionally, β-catenin, as the nuclear accumulation of the pathway, has been highlighted as a critical trigger of osteoblast differentiation and osteogenesis [15]. A previous report has demonstrated that β-catenin functioned as a novel regulatory factor for directly targeting lncRNA KCNQ1OT1 [16].

On the other hand, resveratrol (RSV; 3,5,4′-Trihydroxystilbene), a natural phytoalexin extracted from the root of veratrum grandiflorum, was widely found in a diversity of plants such as grape, peanut, hellebore and so on. Further, reports have shown that RSV had multiple pharmacological properties including antioxidant [17], anti-inflammation [18], anti-necrosis [19], anti-proliferation [20] and anti-cancer [21]. Moreover, accumulating evidence has indicated bone-protective effects of RSV. For example, Mehdi et al. reported that RSV accelerated osteoblastic differentiation in MSCs via Sirt-1/Runx2 activation [22]. Furthermore, Zhang et al. suggested that RSV abrogated NF-κB signaling induced-inhibition on osteoblastic differentiation of BM-MSCs by downregulating TNF-α [23]. However, little was known about the association of RSV and Wnt/β-catenin pathway in terms of osteogenesis.

Therefore, the objective of this study was to explore the underlying effect and the relevant molecular functions of RSV on osteogenic differentiation.

## Methods

### PMMA particles preparation

Purified PMMA particles with the mean particle diameter of 0.330 ± 0.019 μm and 90% of the particles < 1 μm measured using scanning electron microscope were purchased from Polysciences (Philadelphia, PA, USA). The particles were disinfected by torrefaction at 180 °C for 6 h, followed by treatment twice with 70% ethanol at room temperature for 24 h, then washed thrice with sterile phosphate-buffered saline (PBS) and finally desiccated under a bioclean bench. Only endotoxin-free particles were used in forthcoming experiments detected using a Limulus Amoebocyte Lysate assay (Biowhittaker, Walkersville, MD, USA).

### PMMA-induced osteolysis (PIO) animal model

To investigate the role of RSV in osteogenic differentiation and osteolysis, mouse model of PIO was well established as previously described [24]. In short, 30 C57BL/J6 male mice aged 6–8 weeks were divided into three groups as follows: PBS control group (sham, n = 10), PMMA particles in PBS group (PMMA, n = 10) and PMMA particles co-treated with RSV group (PMMA + RSV, n = 10). Mice in each group were anesthetized through single intraperitoneal injection of ketamine (70 mg/kg) and xylazine (5 mg/kg). Centricipital hairs were removed and then a midline incision over cranial bones was cut after disinfection with 5% iodophor. Afterwards, subcutaneous tissues were isolated and cranial periosteum was scraped and then 30 mg PMMA particles evenly were smeared on calvarium followed by full-thickness suture. RSV (Sigma-Aldrich, St. Louis, MO, USA) was orally given 10 mg/kg/day in mice of RSV group while mice in sham group and PMMA group daily received 20 μL injection of PBS. After 2 weeks, all mice were sacrificed and calvarials were collected for RNA and protein extraction. All animal experiments were approved by the Institutional Animal Care and Use Committee at The Affiliated Hospital of Xuzhou Medical University and compliance with the Guide for the Care and Use of Laboratory Animals as well as the Ethics Committee of The Affiliated Hospital of Xuzhou Medical University.

### MMSCs culture and RSV treatment

MMSCs (Cyagen Biosciences Inc., Guangzhou, China) cultured in MSC basal media supplemented with MSC growth supplement (MCGS), 1% l-glutamine and 1% penicillin–streptomycin in a 5% CO_2_ incubator at 37 °C were treated with or without 20 or 40 μM RSV before incubation with 2 mg PMMA particles. MMSCs cultured in medium served as control. After 72 h, the expression levels of KCNQ1OT1, β-catenin, Runx2, Osterix and OCN were determined using qRT-PCR and western blotting. The ALP activity was also measured. In addition, ARS staining was used to examine the differentiation of mMSCs after 21 days.

For further analyzing the biological functions of RSV in osteolysis, mMSCs treated with 40 µM RSV were incubated with or without ICG-001 (10 µM), a selective Wnt/β-catenin inhibitor (Tocris Bioscience, Bristol, UK) for 4 h prior to addition of 2 mg PMMA particles. In addition, mMSCs in media (namely control group) and mMSCs only treated with 2 mg PMMA particles (namely PMMA group) were used as negative controls. Three days later, ALP activity and the expression levels of Runx2, Osterix and OCN at mRNA levels were determined. The differentiation of mMSC was investigated using ARS staining after 21 days.

### Plasmid construction and cell transfection

To explore the latent role of lncRNA KCNQ1OT1 in osteolysis, KCNQ1OT1 sequences were synthesized and subcloned into pCDNA3.1 vector (Invitrogen, Carlsbad, CA, USA) for KCNQ1OT1 overexpression. The pcDNA-KCNQ1OT1 or empty pcDNA vector were transfected into mMSCs seeded in six-well plates at a density of 10^7^ cells/well in MSC basal media containing MCGS at 37 °C with 5% CO_2_ using Lipofectamine 2000 reagent (Invitrogen) according to the manufacturer’s instructions. After 48 h, mMSCs were respectively treated with 2 mg PMMA particles. Additionally, mMSCs in media (control) and mMSCs only treated with 2 mg PMMA particles (PMMA) served as negative controls.

To clarify the interaction between RSV and KCNQ1OT1 in osteolysis, mMSCs were randomly divided into five groups: control, PMMA, PMMA + RSV, PMMA + RSV + sh-NC and PMMA + RSV + sh-KCNQ1OT1. Short-hairpin KCNQ1OT1 (sh-KCNQ1OT1) plasmids and its non-targeting sequence (sh-NC) as a negative control were transfected into mMSCs treated with or without 40 μM RSV in six-well plates with MSC basal media using Lipofectamine 2000 reagent and Opti-MEM I (Invitrogen) when the confluence reached 60%. After 24 h, mMSCs were finally treated with 2 mg PMMA particles.

All mMSCs in above-mentioned groups were collected for ALP activity, qRT-PCR analysis of Runx2, Osterix and OCN expression and ARS staining.

To further understand the regulatory role of KCNQ1OT1 in osteolysis, mMSCs were then randomized to five groups: control, PMMA, PMMA + pcDNA, PMMA + pcDNA-KCNQ1OT1 and PMMA + pcDNA-KCNQ1OT1 + siRNA-β-catenin. Briefly, mMSCs were seeded in antibiotics-free medium in six-well plates at a density of 5 × 10^5^/mL followed by transfection with pcDNA-KCNQ1OT1 or co-transfection with pcDNA-KCNQ1OT1 and siRNA-β-catenin (Thermo Fisher Scientific, Waltham, MA, USA) along with corresponding negative control (pcDNA) using Lipofectamine 2000 (Invitrogen) and then incubated at 37 °C with 5% CO_2_ for 24 h. MMSCs were used to detect the protein levels of β-catenin using western blot, ALP activity and the mRNA expression of Runx2, Osterix and OCN using qRT-PCR as well as mMSCs differentiation using ARS staining.

### RNA pull down assay

The interaction between KCNQ1OT1 and β-catenin was further examined by RNA pull-down using a Pierce Magnetic RNA–Protein Pull-Down Kit (Thermo Fisher Scientific) following the manufacturer’s instructions. The biotin-labeled KCNQ1OT1 was synthesized by transcription using reaction buffer containing Biotin RNA Labeling Mix and T7 RNA polymerase (Roche, Basel, Switzerland), treated with RNase-free DNase I (Roche) for 15 min and purified using an RNeasy Mini Kit (Qiagen, Hilden, Germany). The protein extracts of mMSCs cells were incubated with purified biotin-labeled KCNQ1OT1 and streptavidin agarose magnetic beads (Life Technologies Corporation, Carlsbad, CA, USA), centrifuged at 1500 rpm for 30 min at 4 °C and mixed with PBS and protein loading buffer for 10 min at 98 °C. Eventually, the generated KCNQ1OT1-associated proteins and RNAs were boiled in SDS buffer for detecting β-catenin expressions at protein and RNA levels using IgG antibody as negative control (NC).

### Mouse calvarial model

For in vivo study of RSV, PMMA particle-induced mouse calvarial model was well established. Forty male 6–8 week-old C57BL/J6 mice were averagely distributed into four groups: PBS control (sham, n = 10), PMMA particles in PBS group (vehicle, n = 10), PMMA particles and RSV (RSV group, n = 10) as well as PMMA particles, RSV and ICG-001 (ICG group, n = 10). Mice in sham, vehicle group were daily injected with 20 μL PBS. Besides, mice in RSV and ICG group were treated with RSV at 10 mg/kg/day, while mice in ICG group daily received ICG-001 (10 μL). All mice were sacrificed after 2 week and craniums were separated for ALP activity and the mRNA and protein expressions of β-catenin, Runx2, Osterix and OCN using qRT-PCR.

### QRT-PCR

Total RNA extracted from craniums and mMSCs following transfection was reversely transcribed to cDNA using Prime Script RT Reagent Kit with gDNA Eraser (Takara, Dalian, China). RNA integrity was authenticated by the 260/280 nm ratio using spectrophotometer and electrophoresis method. Ultimately, the relative expression levels of target genes including LncRNA-H19, LncRNA NONHSAT009968, LncRNA KCNQ1OT1, LncRNA ANCR and β-catenin together with Runx2, Osterix and OCN were measured using a SYBR Green Real-Time PCR Master Mix-Plus (Toyobo, Osaka, Japan) on an ABI 7500 Real-Time PCR system (Applied Biosystems, Carlsbad, CA, USA) and analyzed by 2^−ΔΔCt^ method. GAPDH was used as internal controls. Each measurement was performed in triplicate.

### Western blot assay

Total protein was extracted by lysis buffer and isolated using 10% sodium dodecyl sulfate polyacrylamide gel electrophoresis (SDS-PAGE). Subsequently, the protein was transferred to a polyvinylidene fluoride (PVDF) membrane and then blocked with primary anti active β-catenin antibody (Millipore, Bedford, MA, USA) overnight at 4 °C and incubated with anti-mouse horseradish-peroxidase (HRP) conjugated secondary antibody (Cell Signaling Technology, Boston, MA, USA) after washed with Tris-buffered saline containing 10 mM Tris–HCl, 50 mM NaCl and 0.25% Tween 20 at 37 °C for 1 h. Protein quantification was performed using an enhanced chemiluminescence reagent (Beckman Coulter, Brea, CA, USA). GAPDH was used as a loading control.

### ALP activity

ALP activity was considered as a specific marker of osteoblast differentiation which was detected using ALP activity colorimetric assay kit (Sigma-Aldrich). In the beginning, mMSCs incubated with PMMA particles following transfection or treated with different concentration of RSV for 3 days were harvested and rinsed twice with PBS followed by protein extraction using lysis buffer. The remaining supernatant was used to measure total protein content using a BCA protein kit (Pierce Biotechnology, Rockford, IL, USA) in accordance with the manufacturer’s instruction after centrifugation, and the absorption at 405 nm in each well was determined using p-nitrophenyl phosphate (PNPP) substrate (Sigma-Aldrich). However, craniums collected from mouse calvarial model were decalcified using nitric acid-paraformaldehyde solution for 3 days and sectioned followed by dewaxing using gradient ethanol. After that, clear sections were washed twice with PBS and incubated with chromogenic substrates at 37 °C for 1 h. ALP-positive cells were counted under a light microscope.

### ARS staining

Differentiated osteoblasts in vitro could induce cell mineralization after 21 days, which could be specifically stained with ARS. For ARS staining, mMSCs in each group were harvested after 21 days and stained with 40 mM ARS solution (pH 4.2; Sigma) at room temperature for 30 min, followed by lavement with double distilled water thrice and PBS for 15 min finally. Dye absorbance was determined at 570 nm after the addition of 10% cetylpyridinium chloride (pH 7.0; Sigma). Each group was performed in triplicate.

### Statistical analysis

All quantitative data were expressed as mean ± standard deviation (SD) and analyzed using SPSS version 22.0 (SPSS, Chicago, IL, USA). Statistical analyses were performed using two-tail Student’s T test for comparisons between two groups. P value < 0.05 indicated as significantly different.

## Results

### RSV functioned as a modulator in mouse PIO model

**Fig. 1:**
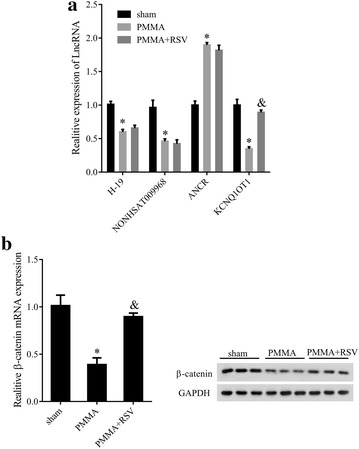
Effect of RSV in PIO mouse model. **a** The relative expression levels of lncRNA H19, NONHSAT009968, ANCR and KCNQ1OT1 quantified by qRT-PCR in mice in the group of sham, PMMA, PMMA + RSV. **b** The mRNA and protein expression levels of β-catenin respectively measured by qRT-PCR and western blotting in mice in the group of sham, PMMA, PMMA + RSV. *P < 0.05 compared with sham group. ^&^P < 0.05 compared with PMMA group

To identify the potential lncRNAs and the role of RSV in osteolysis in vivo, four previously reported lncRNAs, namely H19, NONHSAT009968, ANCR and KCNQ1OT1 were subjected to qRT-PCR analysis for relative expression in mouse model of PIO with or without RSV treatment. As shown in Fig. 1a, compared with the sham group, PMMA treatment led to a decrease in H19, NONHSAT009968 and KCNQ1OT1 expression levels while ANCR was upregulated (P < 0.05), however, RSV treatment increased KCNQ1OT1 expression (P < 0.05), but did not affect H19, NONHSAT009968, ANCR expression levels (P > 0.05). In terms of the remarkable expression alteration, KCNQ1OT1 might be the underlying target of RSV in osteolysis. In the other hand, the expressions of β-catenin at mRNA and protein levels in PMMA group were significantly decreased as compared with the sham group, while RSV treatment reversed the trend (Fig. 1b, P < 0.05). Taken together, RSV upregulated KCNQ1OT1 and β-catenin expression in mouse PIO model.

### RSV alleviated inhibition of osteoblastic differentiation induced by PMMA particles in mMSCs

**Fig. 2:**
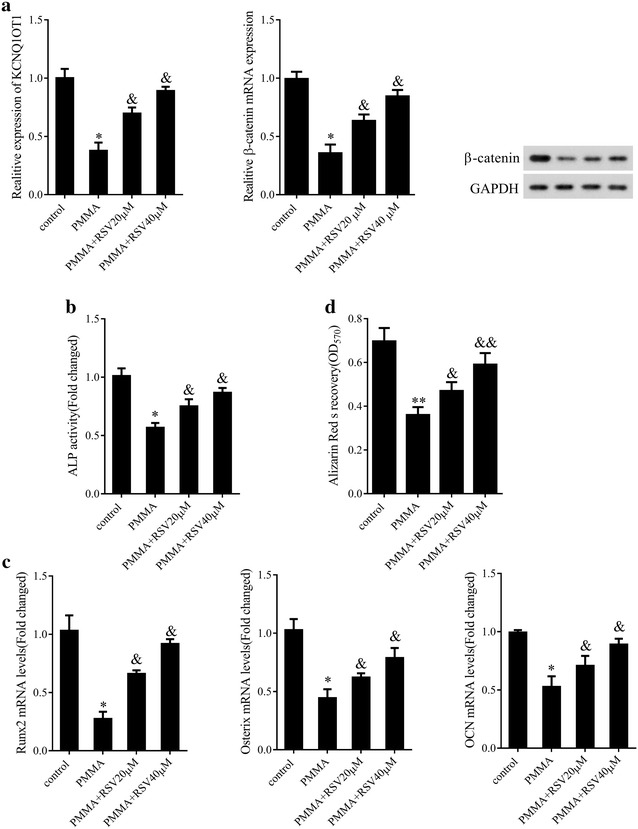
Effect of RSV in mMSCs stimulated with PMMA particles. **a** KCNQ1OT1 relative expression levels and the mRNA and protein expression levels of β-catenin in mMSCs stimulated with PMMA particles treated with or without RSV at different concentration. **b** ALP activity in mMSCs stimulated with PMMA particles treated with or without RSV at different concentration. **c** The mRNA expression levels of Runx2, Osterix and OCN in mMSCs stimulated with PMMA particles treated with or without RSV at different concentration. **d** Osteoblastic differentiation in mMSCs stimulated with PMMA particles treated with or without RSV at different concentration assessed by ARS staining. *P < 0.05 compared with control group. ^&^P < 0.05 compared with PMMA group

Next, we explored the effect of RSV on PIO in vitro. As shown in Fig. 2, PMMA particles observably depressed KCNQ1OT1 expression and the expression of β-catenin at mRNA and protein levels, while RSV treatment led to the opposite effect (Fig. 2a, P < 0.05). Analogously, ALP activity (Fig. 2b, P < 0.05), the mRNA expression of osteoblast-specific transcription factors comprising Runx2, Osterix and OCN (Fig. 2c, P < 0.05) and ARS recovery (Fig. 2d, P < 0.05) which mastered calcium mineralization were markedly reduced in PMMA particles group in comparison with the control group, conversely, RSV treatment inversed the tendency, suggesting that RSV attenuated PIO but promoted osteogenic differentiation of mMSCs in a dose-dependent manner.

### KCNQ1OT1 suppressed PMMA particles induced-inhibition of osteoblastic differentiation of mMSCs

**Fig. 3:**
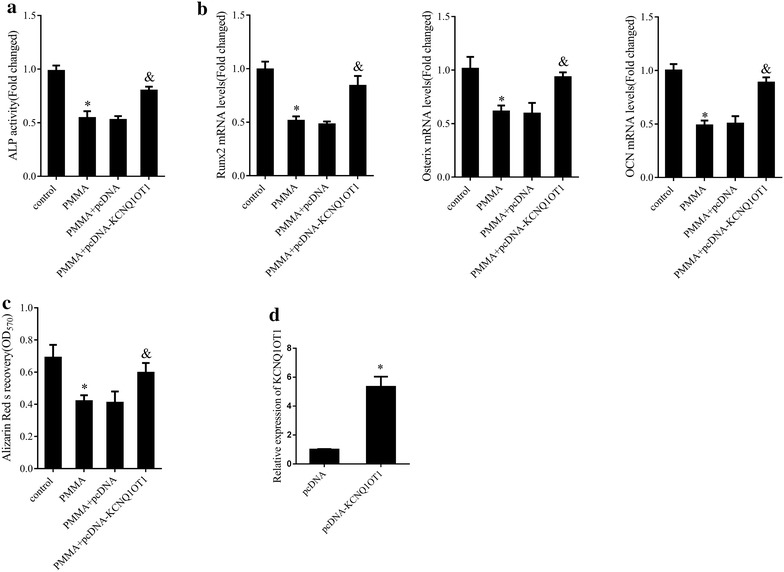
Effect of KCNQ1OT1 in mMSCs stimulated with PMMA particles. **a** ALP activity in mMSCs transfected with pcDNA KCNQ1OT1 or pcDNA before the addition of PMMA particles. **b** The mRNA expression levels of Runx2, Osterix and OCN in mMSCs transfected with pcDNA KCNQ1OT1 or pcDNA before the addition of PMMA particles. **c** Osteoblastic differentiation in mMSCs transfected with pcDNA KCNQ1OT1 or pcDNA before the addition of PMMA particles assessed by ARS staining. **d** The relative expression levels of KCNQ1OT1 in mMSCs transfected with pcDNA KCNQ1OT1 or pcDNA. *P < 0.05 compared with pcDNA-transfected cells. *P < 0.05 compared with control group. ^&^P < 0.05 compared with PMMA + pcDNA group

To evaluate the effect of KCNQ1OT1 in modulating osteogenesis, mMSCs were transfected with pcDNA KCNQ1OT1 for overexpressing KCNQ1OT1 and treated with RSV later. The results showed that PMMA particles treatment signally alleviated ALP activity (Fig. 3a, P < 0.05) and Runx2, Osterix and OCN mRNA expression (Fig. 3b, P < 0.05) as well as calcium mineralization (Fig. 3c, P < 0.05) as compared to the control group. On the contrary, KCNQ1OT1 overexpression enhanced ALP activity (Fig. 3a, P < 0.05), displayed the memorable upregulation of Runx2, Osterix and OCN (Fig. 3b, P < 0.05) and increased the staining density of ARS (Fig. 3c, P < 0.05) even in existence of PMMA particles, since KCNQ1OT1 overexpression upregulated KCNQ1OT1 expression (Fig. 3d, P < 0.05), revealing that KCNQ1OT1 acted as a potential positive regulator of osteoblastic differentiation.

### RSV positively regulated KCNQ1OT1 in PMMA particles-stimulated mMSCs

**Fig. 4:**
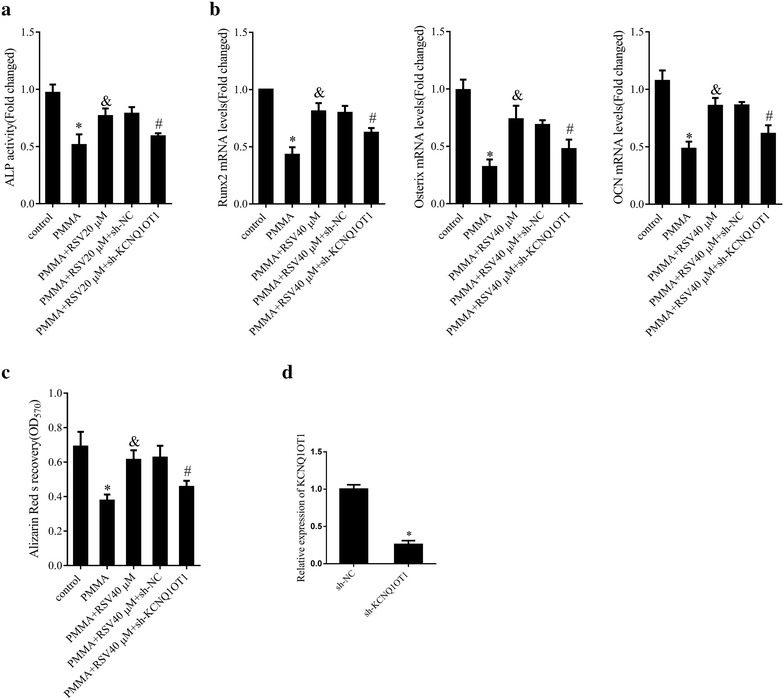
Effect of RSV on KCNQ1OT1 expression. **a** ALP activity in mMSCs transfected with sh-KCNQ1OT1 or sh-NC and treated with 40 μM RSV before the addition of PMMA particles. **b** The mRNA expression levels of Runx2, Osterix and OCN in mMSCs transfected with sh-KCNQ1OT1 or sh-NC and treated with 40 μM RSV before the addition of PMMA particles. **c** Osteoblastic differentiation in mMSCs transfected with sh-KCNQ1OT1 or sh-NC and treated with 40 μM RSV before the addition of PMMA particles assessed by ARS staining. **d** The relative expression levels of KCNQ1OT1 in mMSCs transfected with sh-KCNQ1OT1 or sh-NC. *P < 0.05 compared with sh-NC-transfected cells. *P < 0.05 compared with control group. ^&^P < 0.05 compared with PMMA group. ^#^P < 0.05 compared with PMMA + 40 μM RSV + sh-NC group

We further assessed the interaction between RSV and KCNQ1OT1 in mMSCs stimulated with PMMA particles. Our finding exhibited that ALP activity (Fig. 4a, P < 0.05) and Runx2, Osterix and OCN mRNA expression (Fig. 4b, P < 0.05) as well as the staining density of ARS (Fig. 4c, P < 0.05) of mMSCs treated with RSV were higher than those of mMSCs stimulated with PMMA particles, yet, these trends were reversed by KCNQ1OT1 knockdown, while silencing of KCNQ1OT1 decreased KCNQ1OT1 expression (Fig. 4d, P < 0.05). Thus, it was reasonable to believe that RSV could attenuate particle-induced osteoblastic differentiation inhibition by upregulating KCNQ1OT1.

### RSV activated Wnt/β-catenin signaling in PMMA particles-stimulated mMSCs

**Fig. 5:**
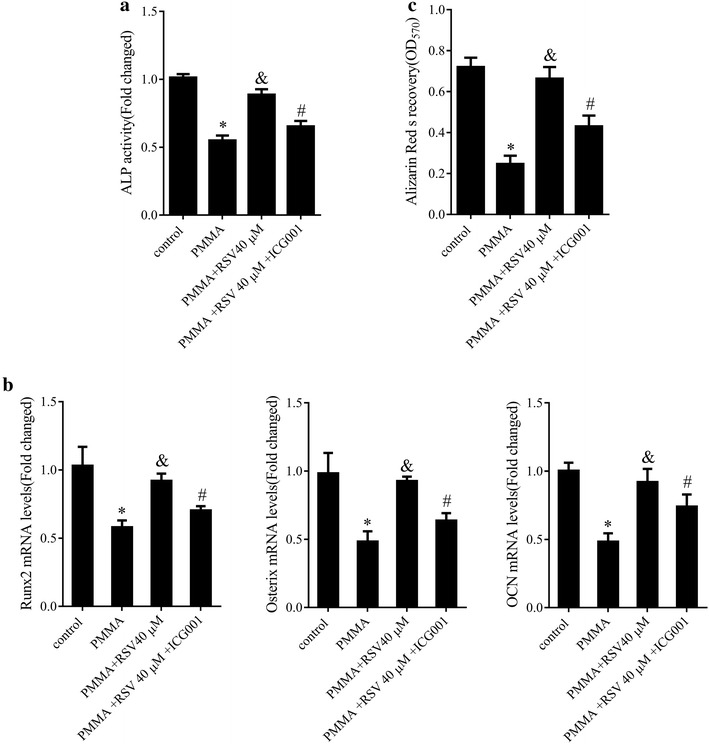
Effect of RSV on Wnt/β-catenin signaling. **a** ALP activity in mMSCs treated with RSV (40 μM) with or without ICG-001 (10 μM) before the addition of PMMA particles. **b** The mRNA expression levels of Runx2, Osterix and OCN in mMSCs treated with RSV (40 μM) with or without ICG-001 (10 μM) before the addition of PMMA particles. **c** Osteoblastic differentiation in mMSCs treated with RSV (40 μM) with or without ICG-001 (10 μM) before the addition of PMMA particles assessed by ARS staining. *P < 0.05 compared with control group. ^&^P < 0.05 compared with PMMA group. ^#^P < 0.05 compared with PMMA + 40 μM RSV group

We also evaluated the molecular mechanism by which RSV attenuated PMMA particles inhibition of mMSCs differentiation. Wnt/β-catenin signaling pathway was reported to play a key role in osteogenesis [13]. In this study, mMSCs were pretreated with RSV (40 μM) and then treated with the Wnt/β-catenin inhibitor, ICG-001 (10 μM) followed by stimulation with PMMA particles. As a result, ICG-001 attenuated RSV-induced pro-differentiation of osteoblast. On the one hand, RSV abolished PMMA particles stimulation-mediated downregulation of ALP activity (Fig. 5a, P < 0.05) and Runx2, Osterix, and OCN mRNA expression (Fig. 5b, P < 0.05) along with the ARS staining density (Fig. 5c, P < 0.05) while ICG-001 notably suppressed RSV-mediated bone-protection. These observations illuminated that RSV alleviated PMMA particles-inhibition of osteoblastic differentiation in mMSCs partly via Wnt/β -catenin signaling pathway activation.

### KCNQ1OT1 alleviated PMMA particles-inhibition of osteoblastic differentiation in mMSCs by regulating β-catenin

**Fig. 6:**
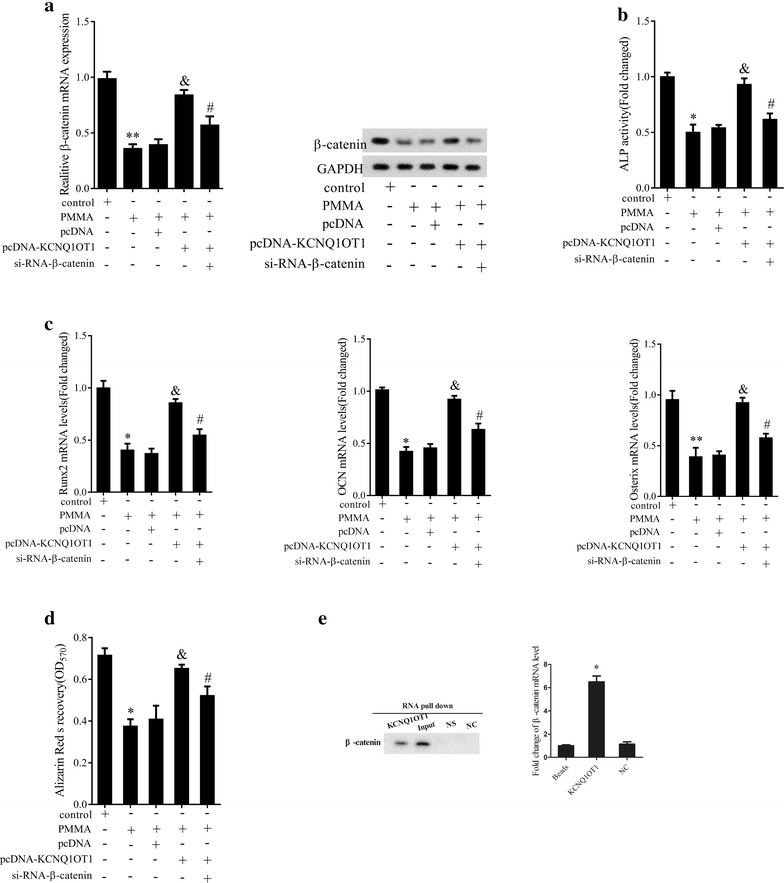
Effect of KCNQ1OT1 on β-catenin in mMSCs differentiation. **a** The mRNA and protein expression levels of β-catenin in mMSCs transfected with pcDNA-KCNQ1OT1, sh-NC or pcDNA-KCNQ1OT1 along with siRNA-β-catenin before the addition of PMMA particles. **b** ALP activity in mMSCs transfected with pcDNA-KCNQ1OT1, sh-NC or pcDNA-KCNQ1OT1 along with siRNA-β-catenin before the addition of PMMA particles. **c** The mRNA expression levels of Runx2, Osterix and OCN in mMSCs transfected with pcDNA-KCNQ1OT1, sh-NC or pcDNA-KCNQ1OT1 and siRNA-β-catenin before the addition of PMMA particles. **d** Osteoblastic differentiation in mMSCs transfected with pcDNA-KCNQ1OT1, sh-NC or pcDNA-KCNQ1OT1 along with siRNA-β-catenin before the addition of PMMA particles. **e** The protein and mRNA levels of β-catenin in KCNQ1OT1 pulled down pellet using IgG antibody as negative control by RNA pull-down assay. *P < 0.05 compared with NC. *P < 0.05 compared with control group. ^&^P < 0.05 compared with PMMA + pcDNA group. ^#^P < 0.05 compared with PMMA + pcDNA-KCNQ1OT1 group

To investigate the regulatory mechanism of KCNQ1OT1 associated with β-catenin, KCNQ1OT1 overexpression and β-catenin knockdown using siRNA were performed in mMSCs. Consistent with previous results, PMMA particles obviously downregulated β-catenin expression at mRNA and protein levels (Fig. 6a, P < 0.05), ALP activity (Fig. 6b, P < 0.05) and Runx2, Osterix, and OCN mRNA expression (Fig. 6c, P < 0.05) together with ARS staining density (Fig. 6d, P < 0.05) as compared to control group, while KCNQ1OT1 overexpression upregulated β-catenin expression (Fig. 6a, P < 0.05), ALP activity (Fig. 6b, P < 0.05) and Runx2, Osterix, and OCN expression (Fig. 6c, P < 0.05) along with ARS staining density (Fig. 6d, P < 0.05). In contrast, β-catenin knockdown inhibited mMSCs differentiation by decreasing β-catenin expression (Fig. 6a, P < 0.05), ALP activity (Fig. 6b, P < 0.05), Runx2, Osterix, OCN expression (Fig. 6c, P < 0.05) and ARS staining density (Fig. 6d, P < 0.05). To further confirm the interaction between KCNQ1OT1 and β-catenin, RNA pull down assay was performed. The expression levels of biotin-labeled β-catenin in KCNQ1OT1 pulled down pellet was higher than that of beads and NC (Fig. 6e, P < 0.05), suggesting the specificity of the interaction between KCNQ1OT1 and β-catenin protein. Collectively, KCNQ1OT1 alleviated PMMA particles-inhibition of osteoblastic differentiation in mMSCs by upregulating β-catenin.

### RSV promoted osteogenic differentiation by activating Wnt/β-catenin signaling in vivo

**Fig. 7:**
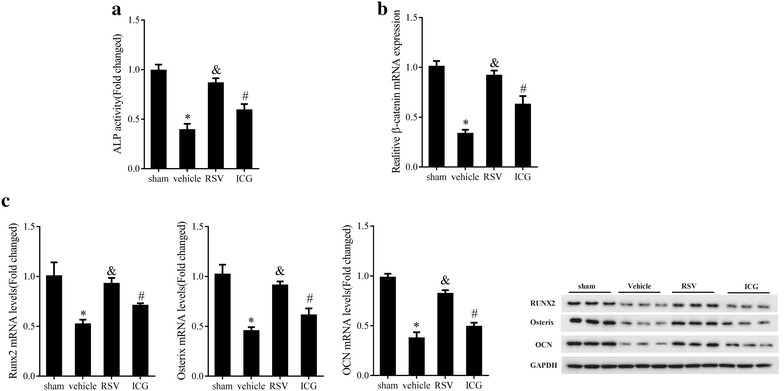
Effect of RSV on activating Wnt/β-catenin signaling in osteogenic differentiation in vivo. **a** ALP activity in calvaria of mouse PIO model pretreated with RSV (10 mg/kg/day) with or without ICG-001 (10 μL). **b** The mRNA expression levels of β-catenin in calvaria of mouse PIO model pretreated with RSV (10 mg/kg/day) with or without ICG-001 (10 μL). **c** The mRNA and protein expression levels of Runx2, Osterix and OCN in calvaria of mouse PIO model pretreated with RSV (10 mg/kg/day) with or without ICG-001 (10 μL). *P < 0.05 compared with sham group. ^&^P < 0.05 compared with vehicle group. ^#^P < 0.05 compared with RSV group

Lastly, we verified whether RSV activated Wnt/β-catenin signaling pathway in vivo. As predicted, ALP activity (Fig. 7a, P < 0.05), β-catenin mRNA expression (Fig. 7b, P < 0.05) and Runx2, Osterix, and OCN expressions at mRNA and protein levels (Fig. 7c, P < 0.05) were dramatically reduced in the vehicle group in comparison with the sham group. Conversely, RSV elevated ALP activity (Fig. 7a, P < 0.05), β-catenin (Fig. 7b, P < 0.05), Runx2, Osterix, and OCN expression (Fig. 7c, P < 0.05), whereas ICG-001 treatment attenuated the effects of RSV (Fig. 7, P < 0.05). Jointly, RSV could promote osteoblastic differentiation via Wnt/β-catenin signaling pathway in mouse PIO model.

## Discussion

RSV was a naturally polyphenolic compound mainly isolated from grape and polygonum cuspidatum which was an effective therapeutic agent for osteoporosis [25] and osteoarthritis [26] and aseptic prosthesis loosening [27]. Previous studies have demonstrated that RSV played an important role in inhibiting osteolysis and promoting osteogenic differentiation [22, 23]. However, the detailed mechanism of RSV in osteogenic differentiation in vitro and in vivo remained indistinct. In this study, RSV upregulated β-catenin and lncRNA KCNQ1OT1 in mouse PIO model, suggesting that lncRNA KCNQ1OT1 and β-catenin were the momentous targets of RSV.

Growing evidence has manifested that osteogenic differentiation was a complicated physiological procedure involving a great deal of regulators containing ALP, Ruxn2, Osterix and OCN [28]. ALP was identified as the paramount marker during the early phase of osteoblastic differentiation [29], while Runx2 and its downstream gene, Osterix were used to evaluate the extent of bone maturation and convert ratio [30]. Meanwhile, OCN mediated terminal osteoblastic differentiation by regulating mineralization [31] which was assessed by ARS staining. Therefore, ALP and Ruxn2/Osterix/OCN were often recognized as primary markers for osteogenic differentiation.

In this study, KCNQ1OT1 and β-catenin expression levels were increased, moreover, ALP activity as well as Ruxn2/Osterix/OCN were obviously elevated after RSV treatment which also promoted the mineralization of mMSCs following PMMA particles stimulation. Besides, KCNQ1OT1 knockdown inhibited ALP activity and Ruxn2/Osterix/OCN as well as mineralization, however, RSV had the adverse effect, indicating that RSV accelerated osteoblastic differentiation in vitro by regulating KCNQ1OT1 and β-catenin.

To further explore the molecular mechanism of KCNQ1OT1 in osteoblastic differentiation, PMMA particles-stimulated mMSCs were transfected with siRNA-β-catenin and/or pcDNA-KCNQ1OT1. The results showed that KCNQ1OT1 overexpression ascended ALP activity and Ruxn2/Osterix/OCN as well as extracellular matrix calcification, while simultaneously overexpression of KCNQ1OT1 and knockdown of β-catenin reversed the trend. These findings elucidated that KCNQ1OT1 alleviated PMMA particles induced-inhibition of osteoblastic differentiation by regulating β-catenin. Since KCNQ1OT1 was proved as the direct target of β-catenin signaling in colorectal cancer, the results indirectly revealed the potential interaction of KCNQ1OT1 and β-catenin.

It was well known that Wnt/β-catenin pathway was an imperative positive regulator of bone homeostasis. In order to further discover the specified mechanisms of RSV in osteoblastic differentiation, its effects involving Wnt/β-catenin pathway were investigated. It turned out that RSV co-treated with ICG-001, a selective Wnt/β-catenin inhibitor decreased the ALP activity and Ruxn2/Osterix/OCN expression along with mineralization in mouse PIO model and mMSCs stimulated with PMMA particles. Consequently, the results confirmed that RSV alleviated PMMA particles-inhibition of osteogenic differentiation by activating Wnt/β-catenin in vivo and in vitro. Analogously, Ji et al. found that RSV exerted the anti-cancer effect via mediating Wnt/β-catenin pathway [16].

In summary, our results uncovered that RSV alleviated PMMA particles-inhibition of osteogenic differentiation in mouse PIO model and mMSCs by upregulating β-catenin and KCNQ1OT1 expression. Meanwhile, KCNQ1OT1 also played similar effect on osteoblastic differentiation by mediating β-catenin. What’s more, RSV promoted osteogenic differentiation and suppressed osteolysis in vivo and in vitro via Wnt/β-catenin activation. Jointly, RSV could relieve osteolysis by promoting osteogenic differentiation through upregulating lncRNA KCNQ1OT1 via Wnt/β-catenin activation. This finding might serve as an essential pre-clinical evidence of RSV for prevention and treatment of bone diseases.
